# Long-term experimental *in situ* farming of *Crambe crambe* (Demospongiae: Poecilosclerida)

**DOI:** 10.7717/peerj.4964

**Published:** 2018-06-13

**Authors:** Andrea Padiglia, Fabio D. Ledda, Bachisio M. Padedda, Roberto Pronzato, Renata Manconi

**Affiliations:** 1 Department for Earth, Environment and Life Sciences, University of Genova, Genova, Italy; 2 Department of Veterinary Medicine, University of Sassari, Sassari, Italy; 3 Centre for Integrative Biology, University of Trento, Trento, Italy; 4 Department of Architecture, Design and Urban Planning, University of Sassari, Sassari, Italy

**Keywords:** Survival and growth, Sponge biomass supply, Marine Protected Area, Seeding season, Substrata, Sardinian Sea, Western Mediterranean, Sustainable bioresources management, Behaviour and life style, Shallow water spongeculture

## Abstract

**Background:**

The marine sponge *Crambe crambe* was chosen as an experimental model of sustainable shallow-water mariculture in the Sardinian Sea (Western Mediterranean) to provide biomass with high potential in applied research.

**Methods:**

Explants were cultured in four long-term experiments (19 and 31 months at ca. 2.5 m depth), to determine the suitability of new culture techniques by testing substrata and seeding time (season), and monitoring survival and growth. Explants were excised and grown in an experimental plant close to the wild donor sponge population. Percentage growth rate (GR%) was measured in terms of surface cover area, and explant survival was monitored *in situ* by means of a digital photo camera.

**Results:**

Explant survival was high throughout the trial, ranging from 78.57% to 92.85% on travertine tiles and from 50% to 71.42% on oyster shells. A few instances of sponge regression were observed. Explant cover area correlated positively with season on two substrata, i.e., tiles and shells. The surface cover area and GR% of explants were measured in the starting phase and monitored up to the end of the trial. High GR% values were observed both on tiles (>21%) and on oyster shells (>15%).

**Discussion:**

The data on the behaviour and life-style of cultured fragments, together with an increase >2,400% in cover area, demonstrate that *in situ* aquaculture is a viable and sustainable method for the shallow-water biomass supply of *Crambe crambe*.

## Introduction

The sustainable exploitation of marine organisms is a key issue for the supply of biomass as a source of bioactive compounds, e.g., in the case of sponges (see Pérez-López et al., 2017). Sponges are key invertebrates in maintaining the biodiversity of benthic communities. The overexploitation of sponge populations could have wide-ranging negative impacts on ecosystems, e.g., biotope architecture and landscape, biodiversity, and trophic and symbiotic relationships (Pronzato, 1999; Wulff, 2006; Bell, 2008; Bell et al., 2015; Wulff, 2017; and references therein).

Several biotechnological approaches have been developed for the production of valuable marine sponge products, including *ex situ* culture (Sipkema et al., 2005) primmorph (Müller et al., 2000; Le Pennec et al., 2003; Valisano et al., 2006; and references therein), and cell and fragment culture (Nickel et al., 2001; Nickel & Brümmer, 2003; Pérez-López et al., 2014; and references therein). Although laboratory experiments on explants are essential to the thorough investigation of sponge biology (e.g., the existence of a developmental growth program and the role of collagen in guiding axial growth; Wanick et al. 2017), ex situ culture has not been considered a feasible means of producing large amounts of biomass (Belarbi et al., 2003; Koopmans, Martens & Wijffels, 2009).

In strategic conservation plans to maintain marine biodiversity, farming sponge explants *in situ* is suggested as one of the most cost-effective and sustainable approaches to producing large amounts of bioactive metabolites (Duckworth & Battershill, 2003a; Page et al., 2005; Pronzato & Manconi, 2008; Murray et al., 2013; Pérez-López et al., 2017; Ternon et al., 2017) and biomaterials (Pronzato et al., 1999; Hoffmann et al., 2003).

Approximately 25 bioactive compounds of sponges and alkaloids from *Crambe crambe* (Schmidt, 1862) were involved in worldwide preclinical pharmacological research conducted in 2012–2013 (Rubiolo et al., 2013; Mayer et al., 2017).

Although protocols have been developed for the short- or medium-term cultivation of several Mediterranean sponge species (Pronzato et al., 1999; Corriero et al., 2004; Ferretti et al., 2009; Osinga et al., 2010; Ledda, Pronzato & Manconi, 2014), few experiments involving the *in situ* and *ex situ* culture of *C. crambe* have been performed (Cebrian et al., 2003; Garcia Camacho et al., 2006; De Caralt et al., 2007; Pérez-López et al., 2014; Ternon et al., 2016; Ternon et al., 2017).

In the present study, *C. crambe* was chosen as a mariculture experimental model for the supply of sponge biomass, owing to its high content of specialized metabolites. New protocols were developed in order to improve sustainable culture techniques of this species in very shallow water (see Pérez-López et al., 2017). Short-, medium-, and long-term experiments focused on constraints such as substrata suitability and thermal stress, which can have positive or negative effects on biomass production and morphofunctional performances under farming conditions. Observations on sponge acclimation, health, survival, growth dynamics, suitable substrates, thermal stress, behaviour, morphotraits and life-style in “captivity” are provided.

## Materials & Methods

### Study area

**Figure 1:**
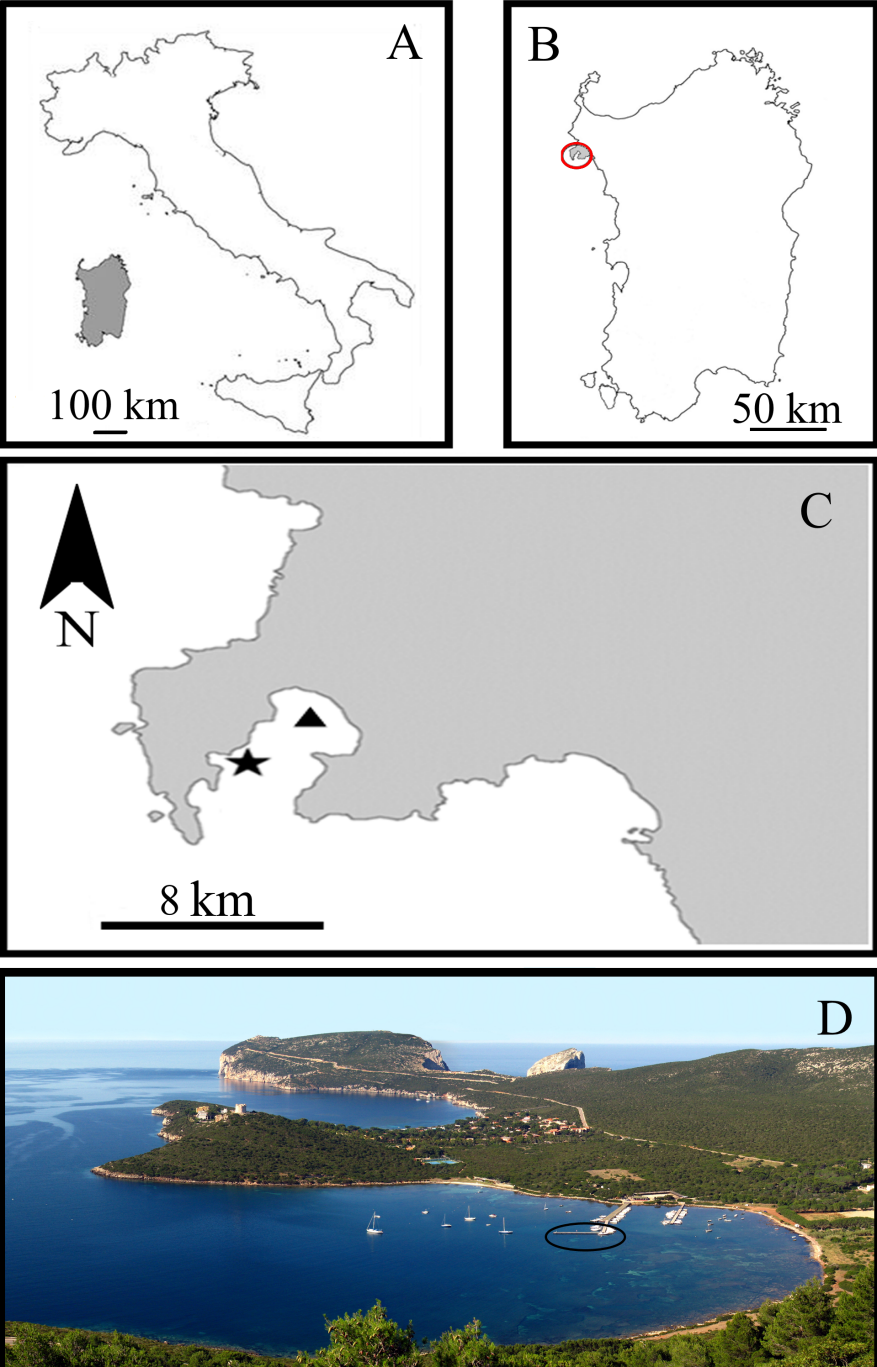
Study area in the Sardinian Sea (Capo Caccia–Isola Piana Marine Protected Area, Western Mediterranean Sea). (A) Sardinia Island (grey) in the Western Mediterranean Sea. (B) Capo Caccia–Isola Piana MPA (grey area within red circle). (C) Sponge culture plant site in Tramariglio Cove (black triangle) and nearby collection site of sponge donors (black star) in Porto Conte Bay. (D) Aerial view of Tramariglio Cove, showing the pier (black circle) to which the sponge culture plant is anchored. Photo credit: the authors.

All experiments were carried out in the Porto Conte Bay, a pristine area of the Northern Sardinian Sea in the C zone of the Capo Caccia –Isola Piana Marine Protected Area (MPA) (Western Mediterranean Sea) (Fig. 1). The sponge-farming plant was opportunistically located in a small marina in Tramariglio Cove (NW Porto Conte Bay, 40°35′33″N, 08°10′12″E), a few kilometres from the sampling site (SE Porto Conte Bay, 40°36′12.61″N, 8°13′6.77″E) on suitable pre-existing submerged man-made structures. The plant modules were anchored to the underwater structure of a pier in very shallow water (2–3 m depth), partially shaded by the pier and sheltered from the prevailing North–Western wind, but occasionally exposed to gales from the South–West.

Prairies of *Posidonia oceanica* (Linnaeus) Delile, 1813 harbouring diversified benthic assemblages are dominant over large extensions of the mainly sandy-silty seabed surrounding the plant in very shallow water (max depth ca. 2.5 m), where patched meadows of the invasive *Caulerpa cylindracea* Sonder, 1845 are also present (Chessa et al., 1989; Gambi et al., 1989; Maj & Taramelli, 1989; Russo et al., 1991; Barberi, Baroli & Cossu, 1995; Gambi et al., 1995). The hydrological characteristics (temperature, salinity) and primary productivity (Chlorophyll *a*) of the Sardinian Sea in the Alghero-Provençal Basin had previously been investigated by Bosc, Bricaud & Antoine (2004) and Olita et al. (2011).

### Experimental design

**Figure 2:**
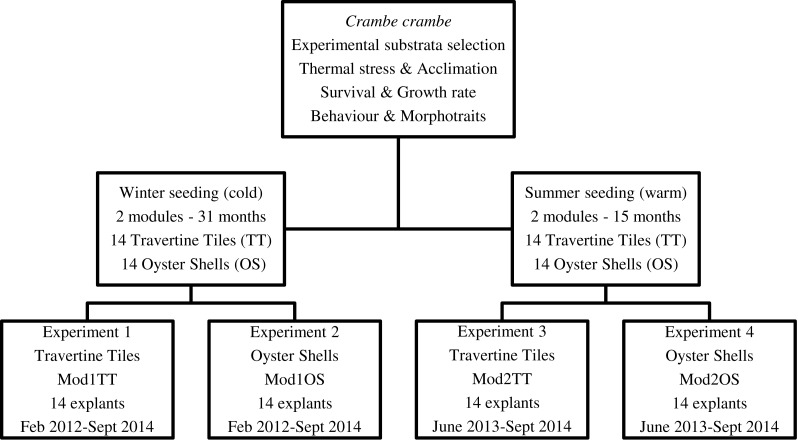
Flow chart of short-, medium-, and long-term experiments on *Crambe crambe* mariculture based on sustainable approaches in very shallow water (Capo Caccia–Isola Piana MPA, Sardinian Sea). Protocols of four experiments were developed in order to focus on environmental constraints: site, depth, water temperature, and substrata type. The temporal dynamics of survival, morphotraits, growth form, cover area, growth rate, behaviour and life style were investigated.

Our target was to optimize *C. crambe* cultivation by identifying suitable conditions: site, depth, method, and water temperature. Four experiments were planned in order to monitor the temporal dynamics of survival, growth form, cover area, and growth rate. To test seasonal thermal stress, the timing of explants—winter (February) vs. summer (June) seeding—was scheduled in order to identify the most suitable seeding time (Fig. 2).

All sponge explants were obtained from 10 wild donors (*n* = 5 summer seeding; *n* = 5 winter seeding), fragmented into 28 explants for each season. For each of the four plant modules, 14 explants of *C. crambe* were seeded in very shallow water (1.5–2.5 m depth) (Fig. 2). Donors were identified following current light-microscopy analysis of the skeletal spicular complement. Taxonomic status was validated on the basis of the description of the family Crambeidae in Systema Porifera (Van Soest, 2002) and the World Porifera Database (Van Soest et al., 2017). Moreover, the metabolome of specimens from the same sampling site was previously analysed (Ternon et al., 2017). Consequently, all explants can be considered to belong to a homogeneous wild population.

### Target species

*Crambe crambe* (Demospongiae: Poecilosclerida: Crambeidae) was selected on account of its ability to produce bioactive compounds, although it is difficult to farm because it needs solid substrata for settlement, owing to its soft, fragile consistency and encrusting growth form. This red sponge species is common and widespread in the entire Mediterranean Sea (Boury-Esnault, 1971; Pulitzer-Finali, 1983; Uriz, Rosell & Martín, 1992; Pansini & Longo, 2008; Van Soest et al., 2017) and the Macaronesian archipelagos (Duran, Giribet & Turon, 2004).

Sexual reproduction in *C. crambe* occurs through internal fertilisation and brooding (viviparity) of lecithotrophic, swimming larvae (large parenchymellas) then released into the water column during July–August in the western Mediterranean populations (Uriz et al., 1998; Uriz, Becerro & Turon, 2001). A high fission rate during asexual reproduction of this species in the wild enhances its rate of spatial expansion (Garrabou & Zabala, 2001).

Toxic compounds are concentrated in the periphery of the sponge body (spherulous cells), protecting *C. crambe* against potential epibionts, endobionts, predators, and competitive neighbours (Uriz et al., 1996), like a chemical shield (see Ternon et al., 2016).

### Sponge sampling

Explants of *C. crambe* were collected from donor specimens by means of SCUBA diving and/or snorkelling at 2–4 m depth in the south-eastern area of the Porto Conte Bay near the farming site (Fig. 1). A significant portion of the wild sponges (donors) were left on their substrata, in order to favour natural regenerative processes.

*C*. *crambe* was scraped from substrata (calcareous rocks, *Spondylus gaederopus* Linnaeus, 1758, and *Arca noae* Linnaeus, 1758) and immediately transferred to the plant. Each sample was cut with scalpels into small replicates of similar size: 3–4 cm in diameter (∼8 cm^2^), thickness <5 mm. Explants were fixed onto two different hard natural substrates. All substrates were suspended in plant modules in accordance with USAMA^®^ patented systems (Pronzato, Manconi & Corriero, 2006).

### Abiotic parameters

The light intensity and water temperature were recorded every 6 h from 2012 to 2014 by means of an underwater HOBO^®^ Data Logger (Onset, MA, USA) installed in the plant. Monthly average values were then calculated.

During the study period, a series of periodic controls in the water column were conducted by means of a multiparametric probe (YSI 6600 V2) to characterize the site. Temperature, pH, salinity, dissolved oxygen and chlorophyll *a* were assessed. Controls were conducted in pre-summer (May 2013 and May 2014) and autumn (October 2013 and November 2014) (Table 1).

**Table 1:** Environmental variables of shallow water in Tramariglio Cove (Capo Caccia –Isola Piana MPA, Sardinian Sea). Mean values recorded in the water column by multiparametric probe (YSI 6600 V2).

Month	Temperature °C	Salinity PSU	Dissolved oxygen %	pH	Chlorophyll *a* µgl^−1^
May 2013	17.8	38.3	111.6	8.22	0.14
Oct 2013	22.5	37.8	92.8	8.18	0.42
May 2014	16.5	38.7	127.1	8.18	0.00
Nov 2014	19.7	38.7	98.1	8.08	0.09

### Substrata tested and seeding seasons

**Figure 3:**
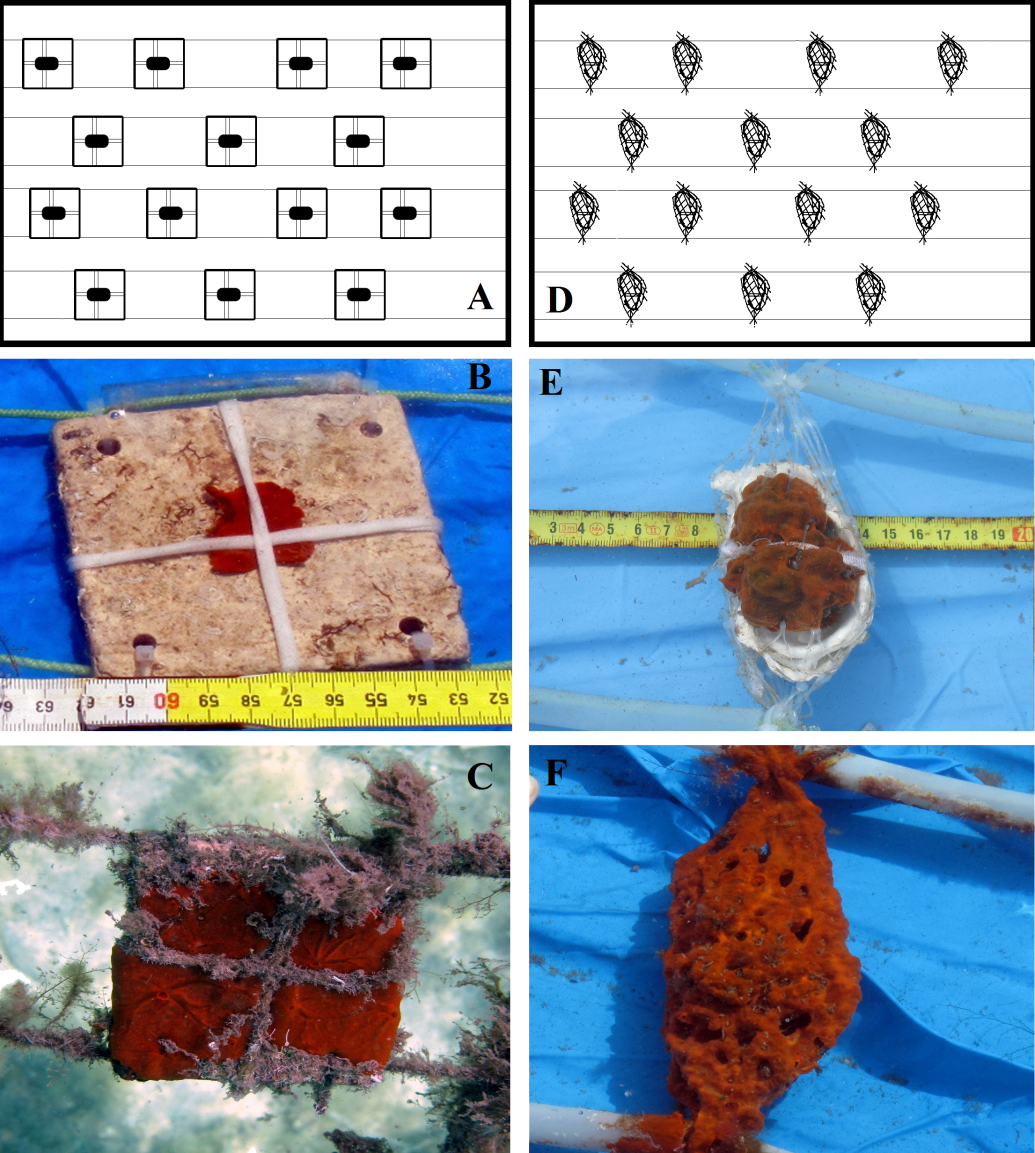
*Crambe crambe* mariculture in shallow-water plant (Capo Caccia–Isola Piana MPA, Sardinian Sea). Schematic drawings of modules with sponges on tested substrata: (A) travertine tiles; (D) oyster shells. Sponge explants settling and growing on experimental substrata: (B) travertine tile in February 2012 vs (C) travertine tile in February 2014; (E) oyster shell in February 2012 vs (F) oyster shell in February 2014. Photo credit: the authors.

To test potentially suitable substrates for *Crambe crambe* settlement, preliminarily experiments were carried out on various kinds of material: simple pockets of soft plastic net, plastic cups, square transparent Perspex/Plexiglas plates, natural stone plates (travertine tiles), and marine biogenic carbonate substrata (oyster shells) (Fig. 2). From among the substrata tested, two natural carbonate substrata were chosen: square travertine tiles and bivalve (oyster) shells (Fig. 3).

Four plant modules (Mod), two for each seeding season, were set up; each module consisted of a square PVC frame, inside which either 14 travertine tiles or 14 oyster shells were suspended; the explants were then seeded onto these substrata (Fig. 3). A code was assigned to each of the four modules (Mod) involved in the four experiments (Exp), i.e., Mod1TT (Exp 1), Mod2TT (Exp 2), Mod1OS (Exp 3), and Mod2OS (Exp 4), denoting both the seeding season (cold-winter = 1; warm-summer = 2) and substrate (Travertine Tile = TT; Oyster Shell = OS) (Fig. 2). The modules were vertically orientated in the water column and anchored to the pier. Each explant was photographed on each occasion of seasonal monitoring.

Empty shells of the commercial oyster *Magallana gigas* (Thunberg, 1793) (previously *Crassostrea gigas*) reared in a Sardinian coastal basin (San Teodoro Lagoon) were recycled. Before being used, the shells were sterilised and maintained in seawater for 48 h.

### Plant modules

Standard USAMA^®^ square modules (60 × 60 cm) made of PVC tubes connected by means of L-shaped joints were used to support the two different natural substrata (TT and OS) for the adhesion and settlement of *C. crambe* explants (Figs. 3A and 3D). Biogenic marine substrates, i.e., Oyster Shells (OS) enclosed singly within a soft net, were fixed at their upper and lower ends to support ropes, which were separated by plastic spacers. Each explant was secured to the shell by means of cotton laces (Figs. 3D–3F). Travertine Tiles (TT) 10 × 10 × 0.5 were anchored to the support ropes by plastic ties threaded through holes drilled in the four corners of each tile (Figs. 3A–3C). Each explant was secured by cotton laces and was partly covered with a fine net to prevent detachment.

### Survival and health

Sponge explants were monitored periodically (3–5 months) to evaluate settlement and adhesion to the substrate, survival, size, growth, and health (presence/absence of necrotic areas); the typical characteristics of the species, i.e., colour, growth form, consistency, and surface traits, were observed in each explant.

### Growth rate

Each explant was photographed alongside a ruler at 3–5-month intervals with a Canon Powershot G-10 camera equipped with a waterproof case. The images were then digitalized to trace the outline of each sponge, and the area in cm^2^ was calculated by means of the software ImageJ 1.47t (National Institutes of Health, Bethesda, MD, USA).

Considering that the encrusting habitus of *C. crambe* shows scant growth in height, sponge growth was monitored in two dimensions as the increase in the covered area of the substrate. The percentage growth rate (GR%) of each explant (used for statistical analyses) was calculated by applying the following formula, adapted for encrusting growth forms from Duckworth & Battershill (2001):

\begin{eqnarray*}\mathrm{GR}\text{%}= \left\{ \frac{[ \frac{({A}_{m}-{A}_{m-1})}{{A}_{m-1}} ]}{n} \right\} \ast 100 \end{eqnarray*}

where *A*_*m*_ = sponge area measured at month *m*, *A*_*m*−1_ = sponge area measured on the previous occasion, and *n* = number of months between one measurement and the next (3–4 months).

GR% was measured during the acclimation phase (four months) and up to the end of experimental period. Data were compared among sponge explants that had settled on the same type of substrate with regard to the seeding season, the time elapsed and the water temperature. The percentage increase was calculated in relation to the area covered at seeding time (Table 2).

**Table 2:** *Crambe crambe in situ* culture in the Sardinian Sea (Tramariglio Cove, Capo Caccia–Isola Piana Marine Protected Area, Western Mediterranean Sea). Dataset of four experiments started in wintertime (February 2012) vs summertime (July 2013). Winter experiments lasted 31 months (Exp 1; Exp 2). Summer experiments lasted 19 months (Exp 3; Exp 4). The area increase value was calculated in relation to AVG cover area at seeding time (winter vs summer). Acclimation Phase (lasting 4 months after seeding) is reported as AP. Months  = m. Minimum and Maximum values of GR cover area increase (see Figs. 5 and 6).

Experiment code	Survival %				AVG cover area cm^2^					Area increase %				Growth rate %		
	AP	12 m	24 m	End	Start	AP	12 m	24 m	End	AP	12 m	24 m	End	AP	Min	Max
Winter																
Exp 1 Mod1TT	100	92.85	92.85	92.85	8.10	11.02	73.13	173.35	202.80	36.00	802.50	2,039.40	2,402.86	5.94	0.36	21.52
Exp 2 Mod1OS	92.85	92.85	78.57	78.57	7.80	8.24	20.51	48.43	58.66	5.05	161.15	517.61	648.02	25.03	0.69	8.70
Summer																
Exp 3 Mod2TT	85.71	57.14	–	50.00	9.47	31.45	48.14	–	47.41	232.09	408.34	–	400.65	22.98	2.52	22.98
Exp 4 Mod2OS	78.57	71.42	–	71.42	6.91	16.10	34.16	–	48.63	132.75	393.88	–	603.04	15.41	0.95	15.41

### Statistical analyses

Repeated-measures analysis of variance (rANOVA) was performed in order to assess the significance of the effect of seeding time (winter vs summer) by comparing cover area and growth rate between pairs of experiments with the same substrate (i.e., travertine tiles Exp 1 vs Exp 3, and oyster shells Exp 2 vs Exp 4) in five successive controls 4/5 months apart. Effects were considered significant for values *p* < 0.05. All data were logarithmically [ln(*x* + 1)] transformed to comply with the assumptions of ANOVA: normal distribution (Shapiro–Wilk test) and homogeneity of variance (Levene’s test). All statistical analyses were performed by means of XLSTAT software (Addinsoft, 2010).

## Results

All the explants responded positively to the plant types and to the new micro-habitat, displaying high survival values and a positive trend in growth rate on both substratum types and in both thermal conditions of seeding. All values related to the acclimation phase refer to 4 months after seeding.

### Growth and survival

#### Acclimation phase

**Figure 4:**
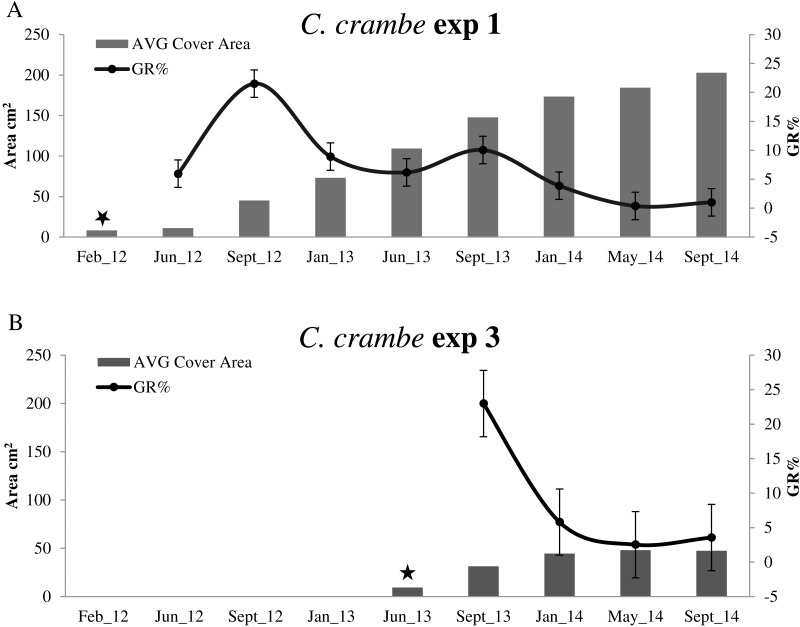
Growth trends in *Crambe crambe* shallow-water mariculture on Travertine Tiles (TT) (Capo Caccia–Isola Piana MPA, Sardinian Sea). Comparison of sponge explants AVG cover area (cm^2^) and Percentage Growth Rate (%); (A) Experiment 1 in Mod1TT (winter seeding, cold water); (B) Experiment 3 in Mod2TT (summer seeding, warm water). Seeding time is indicated by stars.

**Figure 5:**
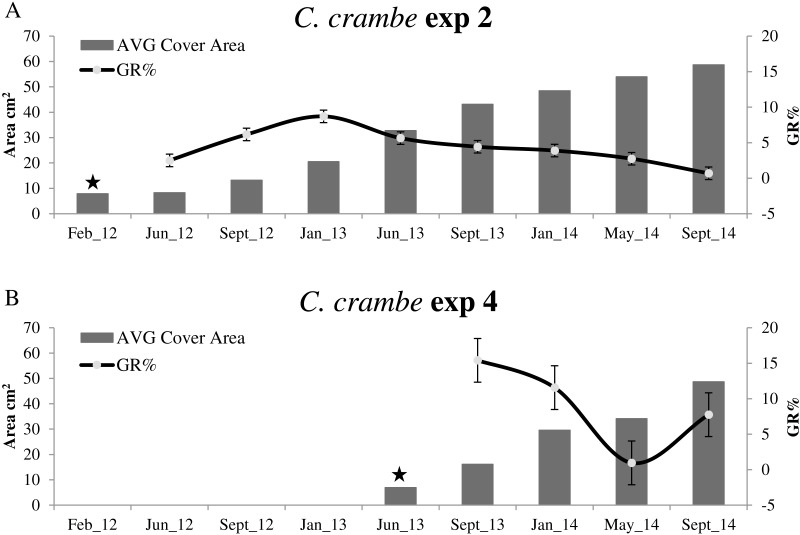
Growth trends in *Crambe crambe* shallow-water mariculture on Oyster Shells (OS) (Capo Caccia–Isola Piana MPA, Sardinian Sea). Comparison of sponge explants AVG cover area (cm^2^) and Percentage Growth Rate (GR%). (A) Experiment 2 in Mod1OS (winter seeding, cold water); (B) Experiment 4 in Mod2OS (summer seeding, warm water). Seeding time is indicated by stars.

Sponge seeding in the two seasons resulted in different growth rate percentages during the 4-month acclimation phases. Regarding the winter seeding, GR% ranged from ca. 5.95% on travertine tiles (Exp 1; Mod1TT) to ca. 25% on shells (Exp 2; Mod1OS) (Figs. 4A and 5A; Table 2). As for the summer seeding, GR% ranged from ca. 22.98% on tiles (Exp 3; Mod2TT) to ca. 15.40% on shells (Exp 4; Mod2OS) (Figs. 4B and 5B; Table 2).

#### Winter seeding

The highest GR% values recorded in experiments on winter seeding were 21.5% (June–September 2012) in Mod1TT (Exp 1) and 8.7% (September 2012–January 2013) in Mod1OS (Exp 2). The lowest values were 0.36% in January–May 2014, after 28 months, in Mod1TT, and 0.69% in May-September 2014, after 31 months, in Mod1OS (Figs. 4A and 5A; Table 2).

#### Summer seeding

Concerning summer seeding, the highest GR% values were recorded in both experiments during the acclimation phase (June –September 2013): 22.98% in Mod2TT (Exp 3) and 15.40% in Mod2OS. The lowest values were 2.52% in Mod2TT in January–May 2014, after 15 months, and 0.95% in May–September 2014, after 19 months, in Mod2OS (Figs. 4B and 5B; Table 2).

#### Long-term dynamics

After 31 months, the average (AVG) cover area had increased by ca. 2,403%, from 8.10 cm^2^ to 202.8 cm^2^, in experiment 1 on tiles (Mod1TT). In experiment 2, by contrast, the AVG cover area had increased by ca. 648%, from 7.8 cm^2^ to 58.66 cm^2^, on shells (Mod1OS) (Table 2).

In experiments 1 and 2, *C. crambe* began to colonize the backside of the substrata after 24 months; both the front and back cover area values were therefore considered for each explant for the last year (2014). In experiments 3 and 4, after 19 months the AVG cover area had increased by ca. 400%, from 9.47 cm^2^to 47.41 cm^2^, on tiles (Exp 3; Mod2TT) and by ca. 603%, from 6.91 to 48.63 cm^2^, on shells (Exp 4; Mod2OS).

**Figure 6:**
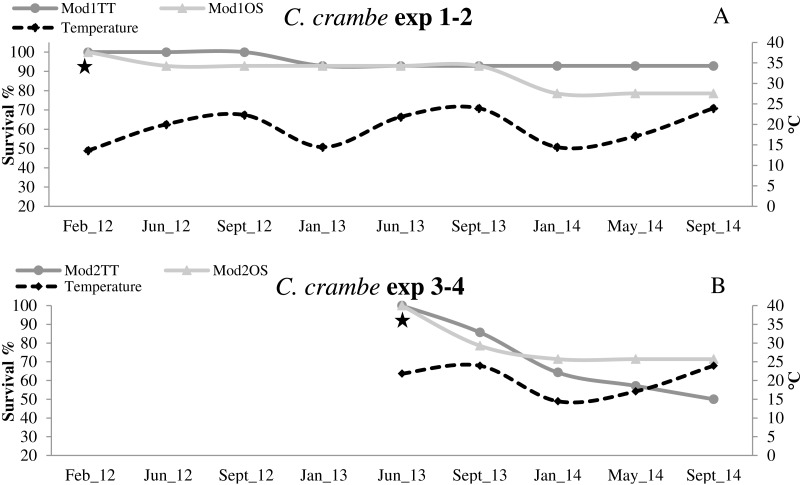
Survival trends in *Crambe crambe* shallow-water mariculture on Travertine Tiles (TT) vs Oyster Shells (OS) (Capo Caccia–Isola Piana MPA, Sardinian Sea). Survival of sponge explants and water temperature trend compared in all four experiments. (A) Experiments 1–2 (winter seeding, cold water); (B) Experiments 3–4 summer seeding (warm water). Seeding time is indicated by stars.

High survival values were recorded in sponges seeded in winter in Mod1TT (Exp 1); after the acclimation phase, survival was 100%, declining to 92.85% after 31 months. In Mod1OS (Exp 2), survival after the acclimation phase was 92.85%, and declined to 78.57% after 31 months (Fig. 6A; Table 2). By contrast, in sponges seeded in summer, survival in Mod2TT was 85.71% during the acclimation phase and 50% after 19 months; in Mod2OS, survival was 78.57% during the acclimation phase and 71.42% after 19 months (Fig. 6B; Table 2).

#### Seeding season

No significant effect of the seeding season (winter vs summer acclimation) was observed on considering cover area data in experiments conducted both on travertine tiles (Exp 1 vs Exp 3; rANOVA, *F* = 2.672, *p* = 0.114%) and on oyster shells (Exp 2 vs Exp 4; rANOVA, *F* = 0.003, *p* = 0.960%).

Regarding growth rate data, a significant effect of the seeding season was observed only in experiments on travertine tiles (Exp 1 vs Exp 3; rANOVA, *F* = 7.761, *p* = 0.10%). No significant effects were seen in experiments on oyster shells (Exp 2 vs Exp 4; rANOVA, *F* = 0.736, *p* = 0.401%).

#### Morphofunctional traits and behaviour

Light microscopy analysis of skeletal morphotraits of sponge samples revealed a spicular complement consisting exclusively of two categories of styles, namely tylostyles and subtylostyles of 230–280 × 3.5–5 µm. This dimensional range matches those reported in the literature (see Rützler, 1965). By contrast the wild population of *C. crambe* at Porto Conte Bay and the cultured sponges at Tramariglio Cove do not have chelae as microscleres.

The reproductive timing of sponges in our experiments was synchronous with that of wild populations, as suggested by the presence of brooded, subspherical, orange larvae in the choanosome during the late spring; this is also in agreement with Becerro, Uriz & Turon (1997).

### Abiotic parameters and site characterisation

The water temperature was calculated by averaging the measurements recorded at the fixed station (1.5 m depth), and varied from a minimum of ca. 14 °C in February to ca. 24 °C from August to October. A minimum value of 7 °C was registered in June 2013 at 6.00 a.m. and a maximum value of ca. 27 °C in August 2013 at 6.00 p.m.

Light intensity recorded at 12 noon throughout the year at 1.5 m depth showed a monthly mean range from ca. 152 to ca. 951 lux. The lowest values were recorded from July to September, probably because the sensor was partly obscured by the flourishing growth of algal mucilage.

The periodic control of the water column parameters (from the surface to ca. 2.50 m depth) highlighted the pristine conditions of the site (Table 1). Water temperature was in line with the seasonality, with no differences along the water column. The high values of salinity (37.8 to 38.6 PSU) indicate the scarcity of continental water inputs and negligible related nutrient loads. pH normally ranged between 8.05 and 8.22. Dissolved Oxygen was markedly above the saturation level on pre-summer control and slightly lower than this threshold during the autumn months. Chlorophyll *a* (<0.5 µg l^−1^), in association with high transparency of the water, was typical of oligotrophic conditions, with low productivity values, in agreement with Olita et al. (2011).

Water monitoring (Directive 2006/7/EC) performed by the Sardinian Environmental Protection Agency (ARPAS) in the four-year period 2013–2016 confirmed that the water in the study area within the Porto Conte Bay in the Marine Protected Area Capo Caccia—Isola Piana was of excellent quality. The ARPAS assessment was based primarily on microbiological parameters and on the evaluation of presence/absence of bituminous residues, glass, plastic or other wastes, phytoplankton blooms, and macro-algae proliferation.

## Discussion

### Substrata for settlement

Substrata were selected after preliminary experiments to identify suitable and/or sustainable materials. Nylon line, which has been used in the farming of other sponge species (Pronzato & Manconi, 2008; Pérez-López et al., 2017) is unsuitable for *C. crambe*, which has a soft, fragile and encrusting growth form. The plastic cups used in preliminary experiments were also deemed unsuitable, as the excessive accumulation of silt clogged the aquiferous system of the explants, causing high mortality. Similarly, transparent Perspex/Plexiglas squares also proved unsuitable (high mortality, low or negative growth), probably because too much light passed through the substratum. Conversely, travertine tiles and oyster shells proved to be optimal for survival and growth, as shown by the present data.

### Behaviour and lifestyle

Field observations confirmed that the explants of *C. crambe* adapted well to their new habitat. Indeed, larval production was seen to be synchronous with that of the wild population of *C. crambe* in the study area; larvae were produced in late spring, in accordance with Becerro, Uriz & Turon (1997).

*C. crambe* seems not to be vulnerable to stress caused by manipulation, as suggested by survival and growth values during acclimation. The sponge displayed marked resilience in response to experimental fragmentation, which is consistent with the processes of fission and fusion that take place during asexual reproduction in the wild, as reported by Garrabou & Zabala (2001).

Allocating the sponge-farming facility to an area close to the wild donor populations enhanced the ability of explants to acclimate rapidly and to re-grow after fragmentation in the new habitat, where the substrata were suspended in the water column and partly shaded by the pier.

With regard to the substrata tested, oyster shells, being natural biogenic marine materials, fit perfectly with the behaviour of *C. crambe* in the Porto Conte Bay. Indeed, the sponge preferentially selects calcareous substrata for larval settlement, such as the surfaces of shells of bivalves (*S. gaederopus*, *A. noae*, *Pinna nobilis* Linnaeus, 1758), and of gastropods (*Hexaplex* spp.), together with coralline algae and crab carapaces (Rützler, 1965; Corriero, Pronzato & Sarà, 1991; R Manconi, pers. obs., 2011). Unfortunately, we were unable to use the shells of these native bivalves as a substrate, notwithstanding their optimal morphotraits (see Marin & López Belluga, 2005), for several reasons: (i) *A. noae* shells are unsuitably small; (ii) these molluscs are not commercialized in Sardinia; (iii) these shells are rarely stranded along the coast; (iv) *P. nobilis*, which has with a suitably large, almost flat shell, is a protected species (2006/105/CE Directive); (v) the low abundance of their populations after several mass mortalities in the past. By contrast, travertine tiles, although man-made, closely mimic natural rocky calcareous marine substrata in terms of both structure and composition.

A peculiar behaviour was displayed by *C. crambe* in the plastic cups tested. Indeed, during the experiments, the sponge explants moved from the inside of the cup (through holes of a few mm in diameter pierced in the bottom) to the outside, where they actively grew on the outer wall until the entire external surface of the cup was encrusted. This ability of sponges to escape unsuitable farming conditions by actively moving on the substratum fits in with the behaviour previously reported by Pronzato (2004) for *Chondrilla nucula* Schmidt, 1862, in similar experimental farming conditions.

### Morphological traits

With regard to growth form and morphofunctional traits, *C. crambe* explants promptly displayed a tendency to assume the typical habitus of wild sponges. Just after fragmentation, the explants had a more or less square flat shape. Subsequently, however, during the early growth phase, a very thin encrusting patina (0.5–1 mm in thickness) expanded from the margins of the explants to colonise the surfaces of both substrata (TT and OS); the body then spread in all directions, reaching a thickness of 0.7–10 mm after some time (e.g., Figs. 3C and 3F). This behaviour was very similar to that seen in the wild population, which usually encrusts and adheres tightly to the irregular surfaces of the shells of living molluscs throughout the *Posidonia* meadows of the bay.

Our cultured sponges were characterised by a spicular complement consisting exclusively of two categories of styles; these were the same as those observed in the wild population of *C. crambe* in the bay, and fit the description provided by Van Soest (2002). In *ex situ* experiments, Maldonado et al. (1999) suggested that *C. crambe* is genetically capable of producing spicule types that are not normally found in all natural populations (i.e., microscleres such as aster-like desmas).

### Thermal acclimation and survival

In all four experiments, after the 4-month acclimation phase, survival was notably high in cold-water seeding conditions (13 °C to 14 °C). The sponges underwent initial stress due to transplantation and seeding in warmer-water seeding conditions (20 °C to 24 °C). Survival data showed that the sponges were still subject to mortality even after the acclimation phase, independently of the seeding season or the substrata. The lower survival values in the explants seeded during the warm season indicate that *C. crambe* seems to be sensitive to warm water during transplantation and acclimation, in agreement with Turon, Becerro & Uriz (1996).

### Cover area and growth dynamics

Our experiments showed that the growth of *C. crambe* on tiles was notably high over the two years up to January 2014 (Fig. 5; Table 2). The growth dynamics was similar in all experiments; initially, a thin film colonised the substratum along the border of each explant, gradually covering the entire available surface of substratum (on both sides) and subsequently increasing in thickness. This phase of colonisation was followed by a phase in which the sponges extended along the support ropes and plastic spacers of the modular plants. Our data show a higher growth rate in the explants seeded on travertine tiles than in those seeded on oysters shells (the highest value was recorded in the acclimation phase on Mod2TT, seeded in summer); this is probably due to the affinity of these encrusting red sponges for relatively corrugated surfaces and porous substrata, rather than the pearly and concave surfaces of shells.

The seeding season seems particularly to influence growth rate; indeed, (rANOVA) a statistically significant association was observed only in experiments on travertine tiles (Exp 1 vs Exp 3), and not on oyster shells. In contrast, no significant relationship (rANOVA) was detected between the cover area and the seeding time in experiments on either tiles or shells, suggesting that the cover area is only constrained by seasonal cycles and time-frame.

In agreement with Turon, Becerro & Uriz (1996), our results show that farmed *C. crambe* explants follow a seasonal trend, i.e., sponges grow faster during the late spring and summer, concomitantly with larval release, while growth is slower but constant in winter, until all the available substratum is covered. These findings are in agreement with data recorded in this species under natural conditions (Turon, Tarjuelo & Uriz, 1998; Garrabou & Zabala, 2001), or in farming experiments involving other species (Duckworth & Battershill, 2003b). Seasonal growth differences have been reported for sponge typically dwelling in shallow water characterised by fluctuating conditions (Lewandrowski & Fell, 1981; Barthel, 1986).

The two-year growth rate of *C. crambe* recorded in the wild by Turon, Tarjuelo & Uriz (1998) was of an average size increase of about 2/5 times in 26 months, whereas in our farming experiments mean size increased about 25 times in 31 months (starting from already-settled fragments). Indeed, it is well known that wild sponges are constrained by substrate competition with other benthic species inhabiting hard substrata (Rützler, 1970). The suitability of the substrata selected and the scant spatial competition contributed to the high growth rate in the Sardinian plant.

High intra-population (farmed sponges) variability in growth rate percentage was observed during our study. Indeed, explants displayed a GR% range from ca. −6% to ca. 29% in September 2012 on Mod1TT; this pattern is reported to be typical of sponges (Ayling, 1983; Todd & Turner, 1988; Stocker, 1991; Turon & Becerro, 1992; Turon, Tarjuelo & Uriz, 1998; Ferretti et al., 2009), which display a wide range of non-synchronous behaviour, i.e., precocious or tardy growth. In our experiments, the data on body size increase were in accordance with high weight increase values (higher than 1000% over the initial weight in ca. 22/45 days), as also reported for *ex situ* explants of *C. crambe* (Belarbi et al., 2003).

## Conclusions

Sponge mariculture and biomass production constitutes a living laboratory for the rational management, conservation and monitoring of marine benthic bioresources. Our long-term *in situ* cultivation experiments supported investigations into the behaviour and strategies of adaptation of *C. crambe* to seasonal, climatic and ecological fluctuations in the pluri-annual cycle by observing quite “pure phenomena” while avoiding intra- and inter-species competition. Technical approaches were improved in order to fit the morpho-, eco- and etho-logical traits of this target species, e.g., the availability of a suitable, natural, hard biogenic substratum for sponges that display a typical thin, fragile, encrusting habitus. The Tramariglio Cove within the Capo Caccia—Isola Piana MPA is an ideal environment for sustainable sponge farming. Indeed, the sustainability of sea-based sponge culture at Tramariglio (with *Sarcotragus spinosulus* as target species) was recently tested by the life-cycle assessment (LCA) approach, which utilises a systematic set of procedures for compiling and examining the inputs and outputs of materials and energy and the associated environmental impacts directly attributable to the functioning of a product or service system throughout its life cycle (see Pérez-López et al., 2017).

**Figure 7:**
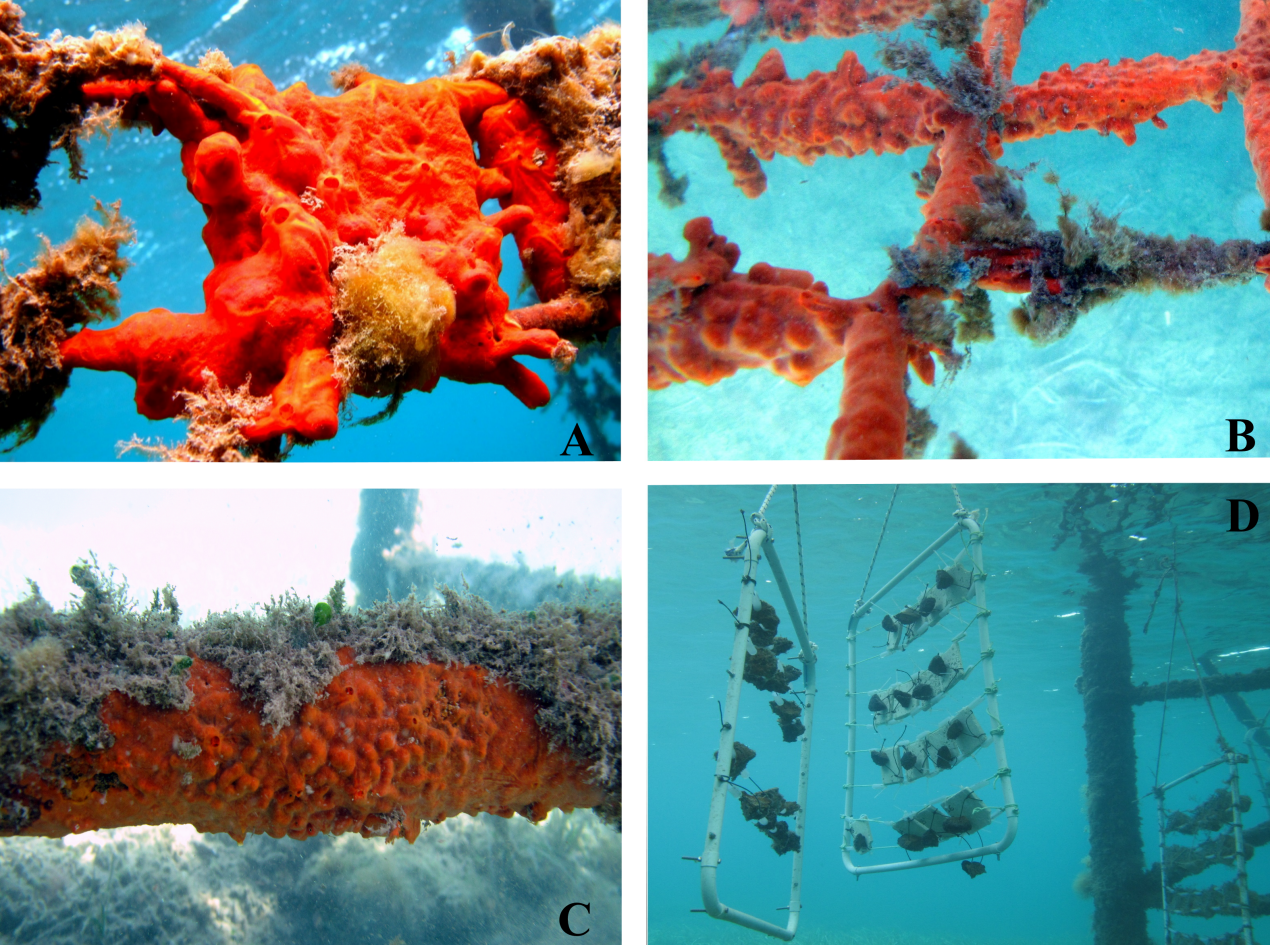
Underwater views of the shallow-water sponge-farming plant in Tramariglio Cove (Capo Caccia–Isola Piana MPA, Sardinian Sea). (A–B) *Crambe crambe* explants entirely covering a travertine tile (A) and oyster shells (B) two years after seeding. This phase of colonisation is characterised by sponges actively growing and moving on all frames of the plant modules (e.g., ropes and plastic spacers). (C) Large specimen of *C. crambe* settled on the pier structure allegedly by means of propagules from the plant. (D) Farming modules anchored by ropes to the pier. Photo credit: the authors.

Explant survival is usually high, and in most cases a long-term healthy growth phase occurs, during which both sexual and asexual reproductive phases can be observed. Moreover, *C. crambe* is particularly adapted to farming in very shallow water (i.e., ca. 2.5 m depth) (Fig. 7).

All present low-tech experiments that use recycled and/or natural substrata for sponge settling are in agreement with sustainable approaches (see Pérez-López et al., 2017). They also avoid potential constraints imposed by artificial materials used in farming, i.e., an aggressive response to chemicals in the materials (Duckworth & Battershill, 2003b). An added value is that these filter feeders intensively farmed *in situ* are able to retain and recycle particulate and dissolved organic matter in the water column (see Ledda, Pronzato & Manconi, 2014).

The occurrence of sexual reproduction also highlights the potential of conserving *C. crambe* by restocking coastal populations with released larvae and asexual propagules from farmed sponges, as previously suggested for other sponge species (Pronzato, 1999; Pronzato et al., 1999; Scalera Liaci et al., 1999; Corriero et al., 2004; Mercurio et al., 2004; Pronzato, 2004; Pronzato & Manconi, 2008). This is particularly true if we consider that one of the preferred substrata of *C. crambe* in the shallow waters of the western Mediterranean consists of shells of living bivalve molluscs, e.g., *S. gaederopus*, *A. noae*, and *P. nobilis*, which have been hit by massive mortality and the disappearance of many populations in recent decades (Meinesz & Mercier, 1983).

Our results are in agreement with those of several authors, who have claimed that the success of farming *in situ* is affected by the seeding season, hydrological conditions, depth, light and location (Wilkinson & Vacelet, 1979; Duckworth, Battershill & Bergquist, 1997; Turon, Tarjuelo & Uriz, 1998; Van Treeck et al., 2003; Duckworth, Battershill & Schiel, 2004). However, Ternon et al. (2017) demonstrated that the production of guanidine alkaloids by *C. crambe* is not constrained by *in situ* farming conditions.

The Sardinian pilot plant was of small size, as the main aim of our study was to identify suitable substrata. The next step will be to increase the size and number of modular structures and calcareous substrata, in order to assess the feasibility of the large-scale biomass production of *C. crambe* for commercial purposes. Indeed, our experiments show that it is possible to renew sponge biomass production in an annual cycle by means of new seeding through the fragmentation of explants from the crop of the same farming plant.

Farming *C. crambe* as a source of bioactive compounds will probably support the supply of marine pharmaceuticals (Mayer et al., 2017) with potential applications for the therapy of cancer and other diseases (El-Demerdash et al., 2018). Moreover, it has a low environmental impact and increases ecosystem services without affecting wild populations (Pronzato & Manconi, 2008). Indeed, the life-cycle assessment previously performed on models of sponge mariculture in the Tramariglio plant for the production of bioactive compounds revealed that the preparation of the crude extract was the main contributor (85–99%) to the environmental burden (Pérez-López et al., 2017). *In situ* sponge culture enables sponges to be grown continuously. Moreover, input requirements are relatively low, as the sponges consume nutrients available in the water column, without raw materials having to be added. At the same time, the bioremediation potential of sponges (filtering capacity; removal of bacterial and organic material) deducts around 5% of the total environmental impact.

##  Supplemental Information

10.7717/peerj.4964/supp-1 Supplemental Information 1 Raw data of *Crambe crambe* mariculture in the Sardinian Sea (S1) Growth rates and mean area throughout the period of study;
(S2) Survival throughout the period of study;
(S3) Water temperature and Lux throughout the period of study.

## Additional Information and Declarations

### Data Availability

The following information was supplied regarding data availability: The raw data are provided in the Supplemental File.
